# Auditory satisfaction of patients fitted with hearing aids in the Brazilian Public Health Service and benefits offered by the hearing aids

**DOI:** 10.5935/1808-8694.20130098

**Published:** 2015-10-08

**Authors:** Deide Paula Costa Braga da Silva, Virgínia Braz da Silva, Fernanda Soares Aurélio

**Affiliations:** a Bachelor's Degree (Speech and Hearing Therapist).; b Specialization (Professor, Speech and Hearing Therapy Program and Graduate Program on Audiology, Faculdade São Lucas. Porto Velho, RO, Brazil).; c MSc (Speech and Hearing Therapist at Clínica Limiar. Professor, Speech and Hearing Therapy Program, Faculdade São Lucas - Porto Velho (RO). PhD student, Health Sciences, University of Brasília (UnB).

**Keywords:** hearing aids, hearing loss, patient satisfaction

## Abstract

The performance of patients fitted with hearing aids dictates the applicable measures to be taken.

**Objective:**

To assess the benefits and degree of satisfaction of adult and elderly patients fitted with hearing aids in a service accredited by the Brazilian Public Health Service.

**Method:**

This descriptive cross-sectional study included 34 individuals with bilateral hearing loss aged 18 and above who had never been offered hearing aids. Scales “Hearing Handicap Inventory for Adults” and “Hearing Handicap Inventory for the Elderly Screening Version” were used to assess the benefits yielded by the hearing aids. Scale “Satisfaction with Amplification in Daily Life” was used to gauge patient satisfaction. The first two were applied on the day the patients were fitted with hearing aids and one month later, whereas the third was applied only one month after the patients had been fitted with the hearing aids.

**Results:**

After the subjects were offered hearing aids, significant reductions were seen in the difficulties they experienced as a consequence of hearing loss. The scores in the Satisfaction with Amplification in Daily Life scale indicated a high degree of satisfaction among patients. These results were not statistically different when gender and age (adult/elderly) subgroups were compared.

**Conclusion:**

The subjects included in the study have benefitted from being fitted with hearing aids and have been very happy with the outcome of the treatment.

## INTRODUCTION

Communication is a vital skill for any individual. It allows the acquisition of knowledge and experience, in addition to playing a key role in social and family interactions. When communication is impaired, the establishment of personal relationships is hampered, leading individuals to isolation and depression^1^.

For a long time, hearing impairment was deemed as an incapacitating disease. A lot has been done in recent years to destigmatize deafness and promote improvements in the quality of life of individuals with hearing loss^2^.

Auditory rehabilitation may be defined as a process designed to allow patients to overcome the barriers to enjoying a fuller participation in daily living activities and mitigate the handicap experienced by the hearing impaired^3^.

The auditory rehabilitation process enables individuals to retrieve their social lives and participate in group activities, thus improving their self-esteem and well-being^4^.

Among other things, the auditory rehabilitation process contemplates the fitting of hearing aids.

These devices have been developed and improved with the purpose of reducing the deleterious effects of hearing loss^5^.

However, the process of selecting and fitting hearing aids can only be effectively completed and produce good outcomes when the patient wears the device properly. And in order for patients to comply with the treatment, they need to see the benefits of wearing hearing aids and be happy with the outcomes produced by the device^6^.

The performance of hearing aids as reported by their users offers guidance to health care professionals as they choose a course of action, in addition to allowing patients to recognize the advantages of wearing the devices over experiencing the hardships of hearing loss, improving compliance to treatment and overall patient satisfaction^6^.

Monitoring patient satisfaction levels and their perceptions while wearing hearing aids is an important step in the assessment of clinical procedures and care quality goals of health services. Once the factors connected to patient satisfaction are identified, health services can significantly improve the effectiveness of the care they provide^7^.

Objective measurements involving formal tasks of speech recognition, or subjective tests based on the benefits perceived by the patients and the difficulties they experience in their daily lives, can be used to gauge the benefits and handicaps patients face while wearing hearing aids^8^.

Self-assessment scales have been used to evaluate treatment plans and the effectiveness of rehabilitation. Effectiveness can be measured as a function of the reduction of disabilities and handicaps, areas of concentration, and patient satisfaction^9^.

The scales most frequently used to assess the benefits yielded by hearing aids are the Hearing Handicap Inventory for Adults (HHIA)^10^ and the Hearing Handicap Inventory for the Elderly Screening Version (HHIE-S)^11^, both developed to evaluate the individual impact of hearing loss. Studies^12^, ^13^ have proven the effectiveness and reliability of these scales.

When patient satisfaction is considered, Satisfaction with Amplification in Daily Life (SADL)^14^, validated in 2000 as a proper instrument to quantify auditory satisfaction^15^, is the most frequently used scale.

Given the facts described above, the, of using these scales lies in the insights they offer into patient perceptions over communication impairments, making it possible to monitor subjects in the long term and to identify their actual auditory needs in addition to the ones observed through routine audiological testing.

In the State Capital of Rondônia, a large portion of the population goes through the process of selecting and being fitted with hearing aids at the Limiar - Hearing Assessment and Rehabilitation Clinic - a private high complexity service affiliated to the Brazilian Public Healthcare System (SUS) as per appointment by Ordinance 589 from October 8, 2004. This facility is a reference center in the State for auditory care and serves local patients along with individuals coming from the country and neighboring States, caring out approximately 70 hearing aid fittings per month, 30 of which in first-time hearing aid users.

Therefore, this study aimed to verify the benefits yielded by hearing aids and the level of satisfaction of adult and elderly individuals fitted with hearing aids in the service mentioned above, in addition to correlating these findings with age (adult and elderly subjects) and gender.

## METHOD

This descriptive cross-sectional study was assessed and approved by the institution's Research Ethics Committee of the São Lucas faculty under the protocol # 23,854. The study was carried out at a high complexity health care facility (Limiar) affiliated to the Brazilian Public Healthcare System (SUS).

Data collection took place in June and July of 2012. All participants signed an informed consent term in which they were explained the study's purposes and method.

Patients had to meet the following enrollment criteria: age of 18 years or older; bilateral hearing loss of any kind and degree; recently fitted with digital bilateral, behind-the-ear, or intracanal hearing aids (new users); agree to join the study and sign an informed consent term. No distinction was made between users of behind-the-ear and intracanal hearing aids, given that a study previously carried out at this center failed to identify differences in the levels of satisfaction of users of either device types^6^.

Subjects with unilateral hearing loss, individuals who had previous experience wearing hearing aids, patients who failed to complete the fitting process, and subjects unwilling to sign the informed consent term were excluded from the study.

The mean number of new hearing aid fittings in adult and elderly patients within a period of two months - 54 - was used as a reference to calculate the size of the sample and the period for which data collection would occur. Considering an error of 10% and a level of confidence of 95%, the size of the sample was set at 35 consecutive patients.

However, one subject did not comply with the fitting process and was excluded from the sample. Therefore, 34 individuals - 20 males and 14 females - were included in the study. Fourteen subjects were adults, with a mean age of 41.7 years, and 20 were elderly, with a mean age of 72.5 years.

The benefits yielded by the hearing aids were measured through the HHIA^10^ and HHIE-S^11^ scales; the first was used to assess adult patients and the second to assess elderly patients.

The HHIA and the HHIE-S are made up of two subscales: Social/Situational and Emotional. The first looks into the impacts of hearing loss upon daily living activities, while the second assesses the attitudes and emotional responses to having hearing loss^8^.

Patients are asked to respond to specific situations and consider whether they represent difficulties to them. The HHIA includes 25 items, 12 in the Social/Situational subscale and 13 in the Emotional subscale.

The score attained in the scale is analyzed as follows: questions answered with a ‘No' are given zero points; ‘Sometimes' is scored as two points; ‘Yes' answers are given four points. The ratings on the HHIA may range from 0%, indicative of no perception of a handicap, to 100% for significant handicap, i.e., lower scores represent fewer difficulties in emotional, social, and situational contexts connected to hearing loss, while higher scores imply more difficulties.

The HHIE-S scale is made of 10 questions. The five questions in the social/situational subscale are designed to identify difficulty-causing situations and find whether hearing loss affects the individual's behavior in the given circumstances. The emotional subscale, also with five questions, is used to assess the attitudes and emotional responses displayed by individuals faced with hearing loss. Scores under 16% indicate absence of handicap; scores between 18% and 42% suggest mild to moderate perception of a handicap; scores above 42% are indicative of severe or significant perception of a handicap.

Patient satisfaction with hearing aids was assessed with the Brazilian Portuguese version of the SADL scale as offered by its authors^16^ with the answer options adapted by Danielli et al.^17^.

The SADL scale includes 15 items divided into four subscales: positive effect (six items connected to acoustic and psychological gains); negative features (three items related to amplification of background noise, feedback, and using a telephone); personal image (three items dealing with cosmetic factors and the stigma associated with wearing hearing aids; and service and cost (three items).

Seven answer options are offered in each question: not at all; a little; somewhat; medium; considerably; greatly; tremendously. The answers are graded based on a seven-point scale, in which the lowest score is one, for ‘not at all', and the highest is seven, for ‘tremendously', indicating lesser and greater levels of satisfaction respectively. Questions 2, 4, 7 and 13 are reversed items, i.e., scores of seven are given to ‘not at all' answers and scores of one are attributed to ‘tremendously' answers.

Patients were advised to assign a score from one to seven to each question.

**Chart 1:**
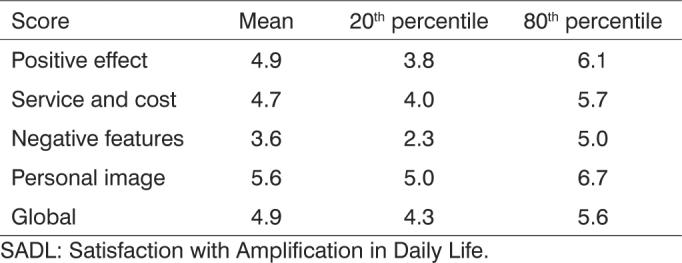
Mean, 20^th^ and 80^th^ percentile SADL global and subscale scores according to the authors of the scale^14^.

Satisfaction was analyzed based on the standards proposed by the authors of the scale^14^. Dissatisfaction was indicated by scores under the 20^th^ percentile; the 80^th^ percentile was used as a reference for satisfied users; patients with scores above the 80^th^ percentile were deemed as very satisfied with their hearing aids (Chart 1).

Data collection was carried out in two stages, one on the day the patients were fitted with hearing aids and another one month after having the devices fitted, when subjects came back to the clinic for functional gain assessment and have their hearing aids reviewed for the first time.

The questions in the scales were read to the subjects by the author in a silent room so as to ensure adequate understanding of the questions and, consequently, answers with proper quality.

The scale used to assess satisfaction levels was applied one month after the subjects had been fitted with the devices, as it takes time for patients to realize the benefits of wearing hearing aids.

The compiled data sets were analyzed with ANOVA statistical tests with significance set at 5% (*p* ≤ 0.05). Assessed variables included age, gender, level of satisfaction, and benefit.

It is worth mentioning that when the HHIA and HHIE-S scales were assessed, weighed values were used to allow the comparison between the scales, given the differences in number of items.

## RESULTS

The answers given to the items in the HHIA and HHIE-S scales applied before and one month after the fitting of hearing aids indicated that the devices provided significant benefit to adult and elderly patients, as illustrated by the reduction in disabilities and auditory handicap posed by hearing loss (Table 1).

**Table 1:** Comparison of findings from HHIA and HHIE-S scales before and one month after hearing aid fitting.

Social/Situational	Adult		Elderly		All		Emotional	Adult		Elderly		All	
	Before	After	Before	After	Before	After		Before	After	Before	After	Before	After
Mean	37	0.29	16.45	0.5	24.91	0.41	Mean	35.57	0.57	14.1	0.3	22.94	0.41
Median	40	0	18	0	20	0	Median	33	0	13	0	18	0
Standard deviation	10.72	0.73	4.71	0.89	12.78	0.82	Standard deviation	11.43	1.45	3.74	0.98	13.21	1.18
Min	14	0	5	0	5	0	Min	18	0	7	0	7	0
Max	48	2	20	2	48	2	Max	52	4	20	4	52	4
N	15	15	21	21	35	35	N	15	15	21	21	35	35
CI	5.43	0.37	2.01	0.38	4.24	0.27	CI	5.78	0.74	1.6	0.42	4.38	0.39
*p*-value	< 0.001*		< 0.001*		< 0.001*		*p*-value	< 0.001*		< 0.001*		< 0.001*	

* Statistical test: ANOVA; level of significance *p* ≤ 0.05. Min: Minimum; Max: Maximum; N: Number of subjects; CI: Confidence Interval; HHIA: Hearing Handicap Inventory for Adults; HHIE-S: Hearing Handicap Inventory for the Elderly Screening Version.

No statistically significant differences were seen in the level of handicap reported by male and female subjects before being fitted with hearing aids. After hearing aid fitting, the difficulties caused by hearing loss were reduced, as shown in the scores under 16% seen in male and female patients. Male patients reported lesser difficulty wearing hearing aids (Table 2).

**Table 2:** Gender comparison of findings from HHIA and HHIE-S scales before and one month after hearing aid fitting.

HHIA/HHIE-S Before	Social/Emotional		Emotional		Global		HHIA/HHIE-S After	Social/Emotional		Emotional		Global	
	F	M	F	M	F	M		F	M	F	M	F	M
Mean (%)	84.3	77.2	75.3	65.7	79.8	71.3	Mean (%)	3.2	0.7	2.7	0.4	2.9	0.6
Median (%)	85.0	86.7	80.0	60.8	80.0	75.5	Median (%)	0	0	0	0	0	0
Standard deviation (%)	15.4	26.9	17.2	21.0	14.8	21.0	Standard deviation (%)	4.6	2.4	5.9	1.7	4.3	1.7
Min (%)	45.8	25.0	38.5	34.6	42.0	30.0	Min (%)	0	0	0	0	0	0
Max (%)	100	100	100	100	100	100	Max (%)	10.0	10.0	20.0	7.7	15.0	6.0
N	14	20	14	20	14	20	N	14	20	14	20	14	20
CI (%)	8.1	11.8	9.0	9.2	7.7	9.2	CI (%)	2.4	1.0	3.1	0.8	2.3	7.0
*p*-value	0.375		0.168		0.204		*p*-value	0.052		0.108		0.032	

Statistical test: ANOVA; level of significance *p* ≤ 0.05. F: Female; M: Male; Min: Minimum; Max: Maximum; N: Number of subjects; CI: Confidence Interval; HHIA: Hearing Handicap Inventory for Adults; HHIE-S: Hearing Handicap Inventory for the Elderly Screening Version.

Statistically significant differences were not seen in the benefits experienced by adult and elderly patients after they started wearing hearing aids (Table 3).

**Table 3:** Age group (adult and elderly patients) comparison of findings from HHIA and HHIE-S scales before and one month after hearing aid fitting.

HHIA/HHIE before	Social		Emotional		Global		HHIA/HHIE after	Social		Emotional		Global	
	Adult	Elderly	Adult	Elderly	Adult	Elderly		Adult	Elderly	Adult	Elderly	Adult	Elderly
Mean (%)	77.1	82.3	68.4	70.5	62.6	76.4	Mean (%)	0.6	2.5	1.1	1.5	0.9	2.0
Median (%)	83.3	90.0	63.5	65.0	77.0	80.0	Median (%)	0	0	0	0	0	0
Standard deviation (%)	22.3	23.5	22.0	18.7	20.9	17.8	Standard deviation (%)	1.5	4.4	2.8	4.9	2.2	3.8
Min (%)	29.2	25.0	34.6	35.0	34.0	30.0	Min (%)	0	0	0	0	0	0
Max (%)	100	100	100	100	98,0	100	Max (%)	4.2	10	7.7	20.0	6.0	15.0
N	14	20	14	20	14	20	N	14	20	14	20	14	20
CI (%)	11.7	10.3	11.5	8.2	10.9	7.8	CI (%)	0.8	1.9	1.5	2.1	1.1	1.7
*p*-value	0.525		0.767		0.572		*p*-value	0.135		0.785		0.316	

Statistical test: ANOVA; Level of significance *p* ≤ 0.05. Min: minimum; Max: maximum; N: number of subjects; CI: confidence interval; HHIA: Hearing Handicap Inventory for Adults; HHIE-S: Hearing Handicap Inventory for the Elderly Screening Version.

A high degree of satisfaction was observed in all subscales and in global scores for the patients included in our study when their scores were compared to the values standardized by the authors of the scales. Mean scores were above the 80^th^ percentile, indicating patients were very satisfied with their hearing aids (Table 4).

**Table 4:** Comparison between global and subscale scores for age groups (adult and elderly patients).

Subscale	Mean		Standard Values	*p*-value
	Adult	Elderly		
Positive effect	6.9	6.7	4.9 (3.8 - 6.1)	0.210
Service and cost	6.8	6.6	4.7 (4.0 - 5.7)	0.269
Negative features	6.8	6.5	3.6 (2.3 - 5.0)	0.249
Personal image	6.7	5.9	5.6 (5.0 - 6.7)	0.078#
Global	6.8	6.4	4.9 (4.3 - 5.6)	0.115

Statistical test: ANOVA; Level of significance *p* ≤ 0.05. ^#^ Tending to significance.

No differences were seen in the degrees of satisfaction reported by adult and elderly subjects, suggesting that both were very satisfied with the performance of their hearing aids (Table 4).

When gender was considered, no statistically significant differences were seen in satisfaction levels, which means that male and female patients were very satisfied with their hearing aids (Table 5).

**Table 5:** Comparison between global and subscale scores for gender.

SADL		Mean	Median	Standard deviation	Min	Max	N	CI	*p*-value
Positive effect	Female	40.07	42.0	4.32	27.0	42.0	14	2.26	0.319
	Male	41.15	42.0	1.73	36.0	42.0	20	0.76	

Service and cost	Female	20.14	21.0	1.88	15.0	21.0	14	0.98	0.942
	Male	20.10	21.0	1.55	15.0	21.0	20	0.68	

Negative features	Female	19.29	21.0	3.54	8.0	21.0	14	1.85	0.265
	Male	20.3	21.0	1.59	14.0	21.0	20	0.70	

Personal image	Female	18.00	21.0	4.96	6.0	21.0	14	2.60	0.494
	Male	19.00	21.0	3.48	10.0	21.0	20	1.52	

Global	Female	97.50	102.5	13.33	58.0	105.0	14	6.98	0.394
	Male	100.55	103.5	7.17	79.0	105.0	20	3.14	

Statistical test: ANOVA; Level of significance *p* ≤ 0.05. SADL: Satisfaction with Amplification in Daily Life; Min: minimum; Max: maximum; N: number of subjects; CI: confidence interval.

## DISCUSSION

Self-assessment scales help speech and hearing therapists monitor the progress of hearing aid fitting and provide insight into the difficulties experienced by individuals wearing hearing aids.

According to the literature, successful device fitting involves multiple aspects, including factors connected to communication and hearing aid user satisfaction^18^.

This study revealed that the significant levels of difficulty related to hearing loss experienced by adult and elderly patients were dramatically reduced after they were fitted with hearing aids. These findings indicate the presence of benefits derived from wearing hearing aids, specifically in regards to social and emotional difficulties, for all patients in the sample, manifested in the form of improved quality of life, a finding consistent with other studies^19^, ^20^ carried out at Brazilian public health care facilities, showing significant reductions on auditory disabilities secondary to the use of hearing aids.

According to some authors^18^, acclimation to hearing aids and consequent improvements in auditory skills may occur as early as one month after device fitting.

A study revealed that objective assessment through speech detection tests showed better outcomes in the months subsequent to hearing aid fitting, in addition to revealing improvements in speech detection skills starting one month after the subjects had been fitted with hearing aids and continuing improvements later on. However, subjective assessment with a scale failed to detect improvements between one and three months after device fitting^21^, which is why the authors of this study decided to assess the benefits of hearing aids only within the first month of use.

No difference was seen in the benefits yielded by the hearing aids when genders were compared. Male and female subjects showed improved communication in daily living activities with the use of hearing aids. The literature^5^ contains reports of auditory recovery in first-time users of hearing aids who had significant improvements in speech after acclimating to the device. However, females were found to have a greater perception of handicap (difficulties experienced due to hearing loss without the use of hearing aids) than males, although this difference was not statistically significant. This finding is in agreement with a study carried out at a medium complexity service accredited by the Brazilian Health System in the State of Paraná, in which female subjects were also found to have a greater perception of handicap, although without statistical significance^22^.

The satisfaction scores of adult and elderly subjects in this study were higher than the scores reported by the authors of the SADL scale^14^ and in other studies in which this instrument was used^6^, ^23^, ^24^, ^25^, ^26^. This difference may be explained by the fact that the subjects in this study were granted hearing aids by the Brazilian Health System and were possibly satisfied with the service they were given.

Scores in the positive effect subscale were higher than the scores published in other studies^6^, ^23^, ^24^, ^25^, ^26^ and than the scores of the 80^th^ percentile of the original study, revealing that the subjects were very satisfied with their hearing aids. The importance of this subscale is confirmed by the improvements in communication and in the quality of the sound delivered by the devices, items strongly correlated with patient satisfaction.

Mean adult and elderly subject scores in the service and cost subscale were above the 80^th^ percentile, indicating levels of satisfaction higher than reported in other studies^6^, ^24^, ^25^, ^26^. The individuals included in this study were given hearing aids by the Brazilian Health System free of charge, which along with good service led to higher mean scores.

Scores on negative features above the 80^th^ percentile were reported in the original study^14^ and by other authors^6^, ^23^, ^24^, ^25^, ^26^, suggesting patients were very satisfied. The literature has correlated lower scores in this subscale with lower levels of satisfaction, with lower scores being usually associated to difficulties while using a telephone^27^.

The mean scores in the personal image subscale were 6.7 for adults and 5.9 for elderly subjects, revealing that adults were very satisfied and elderly individuals satisfied with their personal images as hearing aid users faced with the stigma of wearing hearing aids. Despite the difference in scores, both were higher than the mean values reported by other authors^23^, ^24^, ^25^, ^26^.

A study carried out previously^6^ in the same service as this study looked into the satisfaction levels of adult and elderly patients fitted with hearing aids at different times and found a mean global score of 6.1, suggesting users were quite satisfied with their devices. By its turn, this study included only new hearing aid users and found a higher mean global score (6.8), suggesting the improvement observed after wearing hearing aids for one month generates more satisfaction than when satisfaction is assessed after patients have worn their hearing aids for multiple months.

No difference was seen in the degree of satisfaction of the age groups (adult and elderly patients), indicating patients were more appreciative of their well-being and the quality of life produced as an outcome of wearing hearing aids, rather than sticking to cosmetic concerns.

Along the same lines, subjects of both genders were very satisfied with the use of amplification, as also reported in other studies^6^. Up until recently men resisted more than women to putting hearing aids on, although females were more commonly involved in daily living activities in which communication skills were required more intensely, while men were more concerned with the device's cosmetic impact rather than the quality of life hearing aids could offer. This was not seen in our study, as men and women were very satisfied with their hearing aids and males had a higher mean global score.

The improvement in sound quality and the technology upgrades made to hearing aids are believed to have had significant impact on the increase in the levels of satisfaction observed among hearing aid users, which led to higher levels of device acceptance.

Satisfaction and benefits are apparently related. According to the literature^5^, satisfaction is affected by the user's perception of benefit, comprehending aspects such as the user's expectations, the financial and emotional burden involved, the problems experienced during rehabilitation, and the residual communication difficulties.

## CONCLUSION

This study revealed that adult and elderly patients of both genders had significant reductions in their auditory handicap after they were fitted with hearing aids, in addition to perceiving the benefits and being very satisfied with their hearing aids.
